# A systematic review and meta-analysis of 130,000 individuals shows smoking does not modify the association of *APOE* genotype on risk of coronary heart disease

**DOI:** 10.1016/j.atherosclerosis.2014.07.038

**Published:** 2014-11

**Authors:** Michael V. Holmes, Ruth Frikke-Schmidt, Daniela Melis, Robert Luben, Folkert W. Asselbergs, Jolanda M.A. Boer, Jackie Cooper, Jutta Palmen, Pia Horvat, Jorgen Engmann, Ka-Wah Li, N. Charlotte Onland-Moret, Marten H. Hofker, Meena Kumari, Brendan J. Keating, Jaroslav A. Hubacek, Vera Adamkova, Ruzena Kubinova, Martin Bobak, Kay-Tee Khaw, Børge G. Nordestgaard, Nick Wareham, Steve E. Humphries, Claudia Langenberg, Anne Tybjaerg-Hansen, Philippa J. Talmud

**Affiliations:** a Department of Surgery, Division of Transplantation, Perelman School of Medicine, University of Pennsylvania, Philadelphia, PA, USA; b Department of Epidemiology & Public Health, University College London, London, UK; c Department of Clinical Biochemistry, Rigshospitalet, Copenhagen University Hospital, Copenhagen, Denmark; d The Copenhagen City Heart Study, Frederiksberg Hospital, Copenhagen University Hospital, Copenhagen, Denmark; e The Copenhagen General Population Study, Herlev Hospital, Copenhagen University Hospital, Copenhagen, Denmark; f Faculty of Health and Medical Sciences, University of Copenhagen, Copenhagen, Denmark; g Centre for Cardiovascular Genetics, Institute of Cardiovascular Science, University College London, London, UK; h Department of Public Health and Primary Care, University of Cambridge, Cambridge, UK; i Department of Cardiology, Division Heart & Lungs, University Medical Center, Utrecht, The Netherlands; j Durrer Center for Cardiogenetic Research, ICIN-Netherlands Heart Institute, Utrecht, The Netherlands; k Institute of Cardiovascular Science, faculty of Population Health Sciences, University College London, London, United Kingdom; l Centre for Nutrition, Prevention and Health Services, National Institute for Public Health and the Environment, PO Box 1, 3720 BA Bilthoven, The Netherlands; m Julius Center for Health Sciences and Primary Care, University Medical Center, Utrecht, The Netherlands; n Department of Pediatrics, Molecular Genetics, University Medical Center Groningen and Groningen University, Groningen, The Netherlands; o Center for Experimental Medicine, Institute for Clinical and Experimental Medicine, Videnska 1958/9, Prague 4, 14021, Czech Republic; p National Institute of Public Health, Srobarova 48, 10042 Prague, Czech Republic; q Department of Clinical Biochemistry, Herlev Hospital, Copenhagen University Hospital, Copenhagen, Denmark; r MRC Epidemiology Unit, Institute of Metabolic Science, University of Cambridge, UK

**Keywords:** APOE genotype, Smoking, Coronary heart disease, Gene–environment interaction

## Abstract

**Background:**

Conflicting evidence exists on whether smoking acts as an effect modifier of the association between *APOE* genotype and risk of coronary heart disease (CHD).

**Methods and results:**

We searched PubMed and EMBASE to June 11, 2013 for published studies reporting *APOE* genotype, smoking status and CHD events and added unpublished data from population cohorts. We tested for presence of effect modification by smoking status in the relationship between *APOE* genotype and risk of CHD using likelihood ratio test.

In total 13 studies (including unpublished data from eight cohorts) with 10,134 CHD events in 130,004 individuals of European descent were identified. The odds ratio (OR) for CHD risk from *APOE* genotype (ε4 carriers versus non-carriers) was 1.06 (95% confidence interval (CI): 1.01, 1.12) and for smoking (present vs. past/never smokers) was OR 2.05 (95%CI: 1.95, 2.14). When the association between *APOE* genotype and CHD was stratified by smoking status, compared to non-ε4 carriers, ε4 carriers had an OR of 1.11 (95%CI: 1.02, 1.21) in 28,789 present smokers and an OR of 1.04 (95%CI 0.98, 1.10) in 101,215 previous/never smokers, with no evidence of effect modification (*P*-value for heterogeneity = 0.19). Analysis of pack years in individual participant data of >60,000 with adjustment for cardiovascular traits also failed to identify evidence of effect modification.

**Conclusions:**

In the largest analysis to date, we identified no evidence for effect modification by smoking status in the association between *APOE* genotype and risk of CHD.

## Highlights

• We examined evidence for an interaction between *APOE* genotype, smoking and risk of coronary heart disease.
• This was conducted in the largest meta-analysis of published and unpublished data sets to date (>130,000 individuals).
• Our analysis did not identify evidence of interaction.
• These findings bring into question presence of a clinically meaningful interaction between *APOE* genotype and smoking.

## 1 Introduction

Cardiovascular diseases are the leading cause of death worldwide. Over recent decades, much research attention has focussed on investigating the genetic causes of coronary heart disease (CHD). Despite enormous advances in our understanding of the genetic basis, it is humbling that exposure to tobacco smoke remains a critically important, major, preventable cause of CHD [1].

One of the most widely studied genetic variants in CHD is *APOE*, variation in which encodes the three common isoforms of apolipoprotein E (apoE), ε2, ε3, and ε4, that have important roles in plasma lipid metabolism and transportation [2], [3]. *APOE* variants have a strong and consistent effect on the concentration of plasma lipids and on risk of CHD [4]. ApoE regulates multiple additional metabolic pathways and influences oxidative stress [5]. Previous studies have investigated the potential role of smoking as an effect modifier of the association between genotype and CHD risk [6], [7], [8]. Early studies identified that the association between carriers of the ε4 allele and CHD was greater amongst smokers than non-smokers [6] but this was not replicated in larger studies [7]. However, more recent studies that reported findings in support of effect modification have brought into question the underlying relationship [8], [9].

Effect modification is biologically plausible as *in vitro* studies show that recombinant ApoE ε4 is a poorer anti-oxidant than both ApoE ε2 and ε3 [10], and we previously reported that *APOE* ε4 carriers who smoked had lower anti-oxidant status [6]. Thus it is possible that in the presence of smoking, an impaired anti-oxidant diathesis would result in a greater risk of CHD. If true, the increase in CHD risk caused by smoking should be greater in individuals who carry the ε4 allele compared to individuals who do not carry the ε4 allele. However, an important argument against the plausibility of an anti-oxidant mediated *APOE-*by-smoking interaction is the fact that to date, no randomized trial of an anti-oxidant intervention has shown a reduction in the risk of CHD, which undermines the “oxidation hypothesis” of CHD [11].

It is therefore timely to conduct a large scale, rigorous investigation to address this question. To this end, we conducted a systematic review to identify studies reporting effect modification of *APOE* genotype on risk of CHD by smoking status and supplemented studies that met our inclusion criteria with *de novo* data from large population cohorts. Concern has been raised that the scientific evidence may be interpreted as suggesting that the sequelae of smoking could be less serious in individuals that did not carry the *APOE* ε4 allele [7]. The devastating consequences of smoking to human health is unquestionable, and the purpose of this scientific investigation was not to investigate the potential protection of smoking by certain variants of *APOE* genotype, but rather to investigate the potential for effect modification as a means of understanding the biological processes by which *APOE* increases the risk of CHD.

## 2 Methods

### 2.1 Literature search

We used the PRIMSA statement [12] as a guide and include a completed PRISMA checklist (Supplementary table S1) and flow diagram (Supplementary figure 1). An early analysis plan is included in the Supplementary material.

We searched PubMed and EMBASE from inception to June 11, 2013 for studies that included the keywords “*APOE*”, “genotype”, “smoking” and “CHD”. Full details of the search strategy are provided in the Supplementary material. Eligible studies reported CHD outcomes in relation to *APOE* genotype stratified by smoking status in individuals of European descent. The search was conducted by DM and a random subset of articles was double-checked by MVH. Discrepancies were resolved by consensus. We only included studies that reported the CHD outcome of myocardial infarction (MI) alone or in combination with angina or cardiac interventions (such as revascularization), thus studies reporting stroke or a composite of CHD and stroke were excluded. Furthermore, studies that reported angiographically-determined coronary artery stenosis but not clinical events were also excluded. To minimize human error, DM and MVH conducted data entry separately and checked for concordance between retrieved data.

We updated one previously reported study (NPHSII [6]) for CHD events and supplemented the retrieved articles from the search with eight additional studies: one randomized trial (Thrombosis Prevention Trial [13]), one nested case–control study (EPIC-Netherlands [14]), one cohort (ELSA [15]) and five general population cohorts: Copenhagen City Heart Study (CCHS) [16], Copenhagen General Population Study (CGPS) [17], Czech post-MONICA [18], EPIC-Norfolk [19] and HAPIEE-Czech [20]. Ethics Committees at the contributing centres approved use of new data for updated and unpublished studies.

For all studies, we tested the Hardy Weinberg Equilibrium for the genotypes determined by the two single nucleotide polymorphisms that encode the *APOE* isoforms. For published studies, we noted whether the original report stated presence of effect modification in the *APOE*–CHD relationship by smoking status.

### 2.2 Outcome classification

For the new cohorts (CCHS, CGPS, Czech post-MONICA, EPIC-Netherlands, HAPIEE-Czech), we used ICD codes specific for myocardial infarction (i.e. ICD-8: 410, ICD-9: 410, ICD-10: I21 and ICD-10: I22). For TPT, we used the primary outcome reported in the original randomized trial. Outcome definitions for all studies including those identified from the electronic search and new studies are reported in Table 1.

**Table 1:** Characteristics of the studies included in the analysis.

Ref/study	Study design	Country of origin	Number of study participant	Age, mean (SD) (controls in case–control studies)	Proportion male, % (controls in case-control studies)	Recruitment	Follow-up (years)	CHD outcomes	Outcome ascertainment	*APOE* SNPs in HWE?	Original report stated presence of effect modification?^a^
Published studies identified in the systematic review											
Gustavsson et al. [9]/INTERGENE and SHEEP	Case–control	Sweden	6389	54 (12)	46.2	Cases were patients with first or recurrent CHD and controls were randomly selected from the population	N/A	Acute MI, unstable angina, CHD exacerbations	MI: changes in blood levels of the enzymes CK and LDH, specified ECG-changes and autopsy findings according to the Swedish Association of Cardiologists in 1991	Yes	Yes
Keavney et al. [7]/ISIS	Case–control	UK	7385	46 (10)	67.9	Cases were patients with suspected AMI and controls selected from the relatives and spouses of the case group	N/A	Acute MI	Cardiac enzyme or electrocardiographic criteria, or both	Yes	No
Liu et al. [23]/Physicians' Health Study	Nested case–control	USA	731	60 (9)	100	Male physicians registered with the American Medical Association	12	Fatal and nonfatal MI	WHO criteria for MI. Autopsies and deaths recorded for fatal MI diagnoses.	Yes	No
Talmud et al. [24]/Whitehall II	Prospective cohort	UK	5380	57 (6)	100	Randomly selected among British civil servants	5.8	Fatal/nonfatal MI, angina (definitive or probable)	Fatal MI: national registries; nonfatal MI: MONICA criteria; angina: abnormal investigation such as angiography, exercise electrocardiography, stress imaging, study electrocardiogram or clinical confirmation	Yes	No

Published studies identified in the systematic review updated for incident CHD events											
Humphries et al. [6]/NPHSII	Prospective cohort	UK	2630	55.7 (3.2)	100	General practices, hospital clinics, coroner's offices	>10	Fatal CHD (coronary deaths/fatal MI), nonfatal MI, coronary artery surgery and silent MI	WHO criteria	Yes	Yes

Studies not previously published											
Copenhagen City Heart Study (CCHS)	Prospective cohort	Denmark	8926	55.1(15.1)	44	Population-based	34	Fatal and nonfatal MI	ICD-8: 410; ICD-10: I21 and I22	Yes	N/A
Copenhagen General Population Study (CGPS)	Prospective cohort	Denmark	57,942	57.1(13.3)	44	Population-based	34	Fatal and nonfatal MI	ICD-8: 410; ICD-10: I21 and I22	Yes	N/A
Czech post-MONICA	Prospective cohort	Czech Republic	1875	55.0 (10.3)	45	Population-based	6	Nonfatal MI	ICD-10: I21 and I22	Yes	N/A
ELSA	Cohort	UK	5020	67.5 (9.8)	46	Respondents of Nationwide survey	11	Fatal and nonfatal CHD	Fatal CHD (ICD-10: I20–I25) and self-reported CHD	Yes	N/A
EPIC-Netherlands	Nested case–control	Netherlands	2129	54.1 (10.1)	22	Population-based	13	Fatal and nonfatal MI	ICD-9: 410; ICD-10: I21, I22	Yes	N/A
EPIC-Norfolk	Prospective cohort	UK	22,838	59.2 (9.2)	46	Population-based	10	Fatal and nonfatal MI	ICD-10: I21 and I22	Yes	N/A
HAPIEE-Czech	Prospective cohort	Czech Republic	6256	58.3 (7.1)	46	Population-based	7	Fatal and nonfatal MI	ICD-10: I21 and I22	Yes	N/A
MRC GP Research Framework Investigators, TPT trial [30]	Randomized clinical trial	UK	2503	56.1 (6.7)	100	Hospital clinics	9	Coronary death, fatal and nonfatal MI	WHO criteria	Yes	N/A

CHD, coronary heart disease; CK, creatine kinase; ECG, electrocardiogram; HWE, Hardy Weinberg Equilibrium; ICD, international classification of disease; LDH, lactate dehydrogenase; MI, myocardial infarction; N/A, not applicable; WHO, World Health Organization.

a Effect modification by smoking status in the APOE–CHD relationship.

### 2.3 APOE genotype grouping

We conducted two separate genetic analyses: one simple, and one detailed. For the simple analysis, we stratified individuals into two groups, based on carriage of the ε4 allele of *APOE* genotype: ε4 carriers consisted of *APOE* genotypes ε3/ε4 or ε4/ε4; non-ε4 carriers consisted of *APOE* genotypes ε3/ε3, ε2/ε3 or ε2/ε2. Where possible, ε2/ε4 carriers were excluded by convention [6], [21].

For the detailed genetic analysis, we used the original *APOE* genotype groups and placed them into order according to the previously reported association with CHD [4] (i.e. from lowest to highest CHD risk: ε2/ε2, ε2/ε3, ε3/ε3, ε3/ε4 or ε4/ε4). Treating *APOE* genotype in this fashion yields a monotonic relationship between genotype group and risk of CHD [4].

### 2.4 Analysis

#### 2.4.1 Smoking status: present versus past/never

Using tabulated data, we reconstructed the original data sets with the following variables: *APOE* genotype, smoking status (present, past or never) and CHD status. This enabled us to conduct “quasi-individual participant data” analyses to investigate the association between *APOE* genotype and CHD risk. The NPHSII study was stratified as previous studies have shown heterogeneity according to recruitment centre [22]. For the analyses described below, we used logistic regression with CHD status as the dependent variable; the principle summary measure was therefore an odds ratio (OR). In every analysis, we adjusted for study design using an unordered categorical variable with the “i.” prefix in Stata.

For the simple analysis, we initially tested the univariate association between smoking and *APOE* ε4 genotype carrier status (comparing ε4 carriers to non-carriers) individually with CHD, which served to validate our data set for the analysis of effect modification. Second, we stratified the association between *APOE* genotype and CHD by smoking status (into present vs. previous/never; this grouping was used as it was the most widely-reported in the identified studies and allowed us to include all studies). Finally, we tested for an interaction between *APOE* and smoking by fitting two statistical models: (i) a multivariate model with CHD status as the dependent variable, and smoking status and *APOE* genotype as the independent variables (adjusting for study design), and (ii) a model consisting of the same variables as model (i), but with an interaction term fitted between smoking status and *APOE* genotype. We used the Likelihood ratio test (LRT) to test the null hypothesis that the simpler model lacking the interaction term, i.e. model (i), explained the data better. We used a generous *P*-value threshold (<0.05) from the LRT as evidence against the null hypothesis (of no effect modification). To investigate whether there was a relationship between sample size and evidence for effect modification, we also conducted the LRT for effect modification between *APOE* genotype and smoking status in each study individually, and plotted the *P*-values derived from LRT against sample size, grouped by whether the original publication reported evidence of effect modification. We tested for evidence of a pair-wise correlation between sample size and LRT *P*-value by obtaining the Pearson’s correlation coefficient from the “pwcorr” command in Stata.

In the detailed genetic analysis, we investigated the association between *APOE* genotype with odds of CHD in the three large population cohorts (EPIC-Norfolk, CCHS and CGPS) in which outcomes were ICD codes for myocardial infarction, with detailed information on smoking (present, past, never) and *APOE* genotype. We arranged genotype groups in order according to the reported association with CHD risk in the largest genetic association reported to date [4]. Thus, individuals were assigned a numerical value of 1–5 depending on their *APOE* genotype (1 = ε2/ε2, 2 = ε2/ε3, 3 = ε3/ε3, 4 = ε3/ε4 or 5 = ε4/ε4). In all individuals (irrespective of smoking status), we first obtained the odds ratio of CHD for each individual group by conducting a categorical logistic regression analysis, using the ε3/ε3 genotype as the reference group. We then tested for the presence of a linear association between the *APOE* genotype groups using logistic regression, by treating the *APOE* genotype as a continuous trait (thus the beta coefficient on the log odds scale for this trait was the slope for an incremental increase in *APOE* genotype). Next, we stratified the analysis by smoking status into present, past or never and reconstructed the plots. For each of these three smoking groups, we generated a slope for the association between *APOE* genotype and log odds of CHD. Finally, we tested for a difference in these three slopes (of the linear estimates for *APOE* genotype by smoking status: present, past, never smoking) by fitting an interaction term between *APOE* genotype status and smoking status in the logistic regression model and using likelihood ratio test to investigate whether a simpler model (without the interaction term fitted) better explained the data.

#### 2.4.2 Pack years and adjustment for other cardiovascular risk factors

In addition to the analysis of present vs. past/never smoker in all studies (including published and unpublished data), access to individual participant data in two large population-based studies (Copenhagen General Population Study and Copenhagen City Heart Study) with a pooled sample size of 68,177 facilitated the conduct of a more detailed analysis of smoking phenotype and permitted statistical adjustment for cardiovascular risk factors.

For this, we used pack years, a trait that encompasses both the number of cigarettes smoked per day and the duration of smoking (one pack year is equivalent to 20 cigarettes smoked every day for one year). We conducted logistic regression analyses using pack years as a categorical variable. In detail, the dependent variable was MI, and the independent variables were pack years (treated as an unordered categorical variable with individuals grouped into 0, >0 to <10, ≥10 to <20 and ≥20 pack years), and *APOE* ε4 genotype carrier status (dichotomized into ε4 carriers and non-carriers). This was conducted initially in the two studies separately, unadjusted for any other traits. The model was then repeated with an interaction term fitted between pack years and *APOE* genotype status and the Likelihood ratio test was used to test model fit. We then repeated these analyses with statistical adjustment for the following cardiovascular traits (that could act as confounders): (i) age (grouped into 10-year blocks), (ii) gender, (iii) hypertension (classified as SBP ≥140 mmHg or DBP >90 mmHg) and (iv) type 2 diabetes (T2D, ascertained from the following ICD codes: ICD-8: 250; ICD-10: E11, E13 and E14) to investigate whether an interaction between pack years and *APOE* genotype emerged. Finally, a fully adjusted model was fitted that included all cardiovascular variables.

All analyses were conducted using Stata version 13.1 (StataCorp, College Station, Texas 77845 USA).

## 3 Results

Of the 425 articles retrieved from the search, five studies met our inclusion criteria (Supplementary figure 1) [6], [7], [9], [23], [24]. To this, we updated data from one cohort and added data from eight new studies (Supplementary figure 1). This yielded a total of 13 studies with 10,134 CHD events in 130,004 individuals of European descent of whom 28,789 were present smokers.

Study characteristics including age and gender are provided in Table 1. Twenty-two per cent of individuals in the pooled data set were current smokers and 28% were carriers of the *APOE* ε4 allele (study-level characteristics are reported in Supplementary Table S2). The genotypes of the SNPs comprising the *APOE* isoforms were in Hardy Weinberg Equilibrium in all contributing studies. Two of the five previously published studies reported evidence of effect modification of the association between *APOE* genotype and CHD risk by smoking status [6], [9]. In these two studies, the outcomes were a composite that included angina, silent MI or coronary artery surgery.

### 3.1 Univariate association of APOE genotype and smoking status with CHD

Carriers of the ε4 allele had an OR of CHD of 1.06 (95%CI 1.01, 1.12; *P* = 0.01) compared to individuals who did not carry the ε4 allele. Individuals who currently smoked had an OR of CHD of 2.05 (95%CI: 1.95, 2.14; *P* = 9.7 × 10^−^^196^) compared to individuals that did not currently smoke (including previous or never smokers).

#### 3.1.1 Association of APOE ε4 allele genotype with CHD stratified by smoking status and test for interaction

In analysis of 6148 CHD cases in 101,215 individuals who did not currently smoke, carriers of the ε4 allele had an OR of CHD of 1.04 (95%CI 0.98, 1.10; *P* = 0.25) compared to individuals who did not carry the ε4 allele. In 28,789 current smokers with 3986 CHD events, carriers of the ε4 allele had an OR of CHD of 1.11 (95%CI 1.02, 1.21; *P* = 0.02) compared to individuals who did not carry the ε4 allele.

**Fig. 1:**
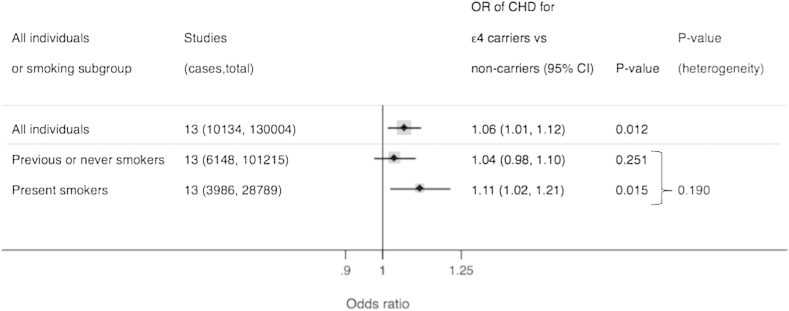
Association between *APOE* genotype and CHD in all individuals and stratified by smoking status. *P*-value for heterogeneity obtained from testing whether an interaction term between *APOE* and smoking represents the data better than no interaction term (tested using likelihood ratio test).

When tested formally using likelihood ratio test, we identified no evidence of an interaction between *APOE* genotype and smoking (Parameter estimate = 0.07; 95%CI: −0.03, 0.17; *χ*^2^ 1.72; (df = 1); *P* = 0.19) (Fig. 1).

**Fig. 2:**
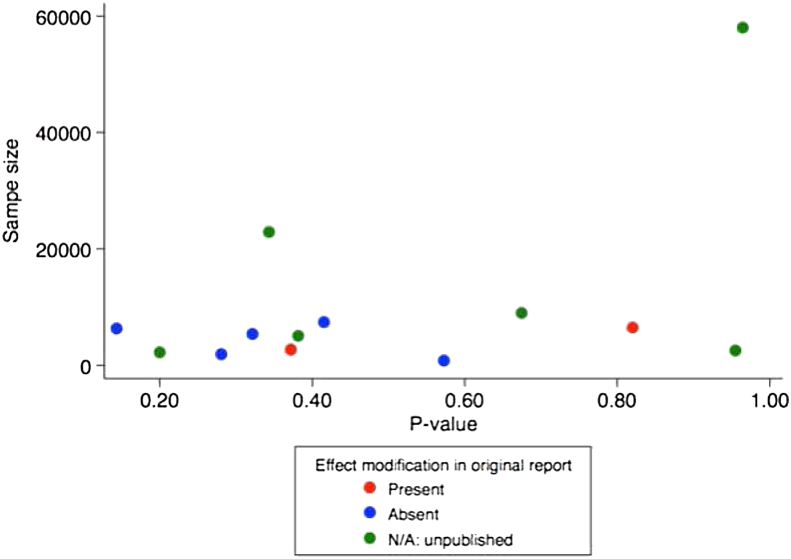
Scatter plot of the *P*-value for interaction and sample size in the 13 studies. *P*-value obtained from testing whether an interaction term between *APOE* and smoking represents the data better than no interaction term (tested using likelihood ratio test). The correlation between *P*-value and sample size was Pearson's *r* = 0.45 (*P*-value for correlation = 0.13).

An investigation into the relationship between sample size and *P*-value for effect modification in each study, categorized according to whether the original publication reported presence of effect modification, did not identify a relationship between sample size and *P*-value for LRT (Pearson's *r* = 0.45; *P*-value for correlation = 0.13). Interestingly, using our analytical approach, we did not reproduce the small *P*-values for effect modification in either of the two previously reported studies, both of which had fewer than 7000 participants. In contrast, the largest study in our analysis, with a sample size of 57,942 (and which used ICD codes specific for MI), had the largest *P*-value for interaction (*P* = 0.96; Fig. 2).

### 3.2 Detailed analysis

**Fig. 3:**
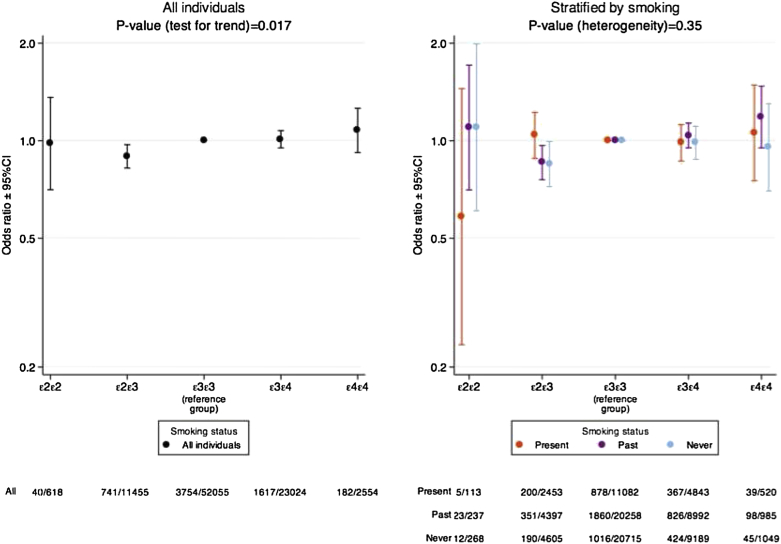
Detailed association between *APOE* genotype and CHD overall (left) and stratified by smoking status (right).

When we investigated the association between *APOE* genotype status and CHD regardless of smoking status, we found strong evidence for a linear association between *APOE* genotype and CHD status when individuals were arranged in the following order: ε2/ε2, ε2/ε3, ε3/ε3, ε3/ε4 or ε4/ε4 with the log odds of CHD increasing by 0.046 (SE 0.019; *P* = 0.017) for each incremental increase in *APOE* genotype status (Fig. 3).

When we stratified this analysis by smoking status into three groups (present, past or never smokers), and tested for evidence of heterogeneity in the association between *APOE* genotype and odds of CHD according to smoking status, we found no evidence to support effect modification (*P* for heterogeneity of slopes by smoking status = 0.35; Fig. 3 and Supplementary Table S3).

#### 3.2.1 Analysis of pack years in two large prospective cohorts with MI outcomes

In 59,349 and 8828 individuals in CGPS and CCHS, respectively, when an interaction term was fitted between *APOE* genotype (ε4 allele carriers vs. non-carriers) and pack years, no evidence for an interaction for risk of MI was identified (*P*-values for interaction = 0.54 and 0.65 for CGPS and CCHS, respectively) (Supplementary Tables S4 and S5). This finding did not alter with subsequent adjustment for age, gender, hypertension or T2D (Supplementary Tables S6 and S7).

## 4 Discussion

This study represents the largest investigation to date into the role of smoking in the association between *APOE* genotype status and risk of CHD. We conducted a systematic review and supplemented identified studies with unpublished and updated data, and found that the association between *APOE* genotype and CHD was not meaningfully different when stratified by smoking status. Our interpretation is that there is no evidence to support the hypothesis that smoking is an effect modifier of the association between *APOE* genotype and CHD events.

In clinical trials, the standard method of testing effect modification is to perform analyses stratified by the potential modifier [25], and investigate for differences between the treatment effects amongst strata. Owing to the random allocation of alleles at gametogenesis, genetic studies share many of the features of randomized trials [26], thus a similar strategy can be used for investigating gene–environment interactions. This can be conducted by comparing summary estimates from meta-analysis stratified by the potential effect modifier, or by re-constructing individual participant data (from summary tabulated data, as we did in this analysis) and conducting a likelihood ratio test with and without an interaction term fitted between the exposure (in this case *APOE* genotype) and potential modifier (smoking status). A large *P*-value on likelihood ratio test (as identified in our analysis) makes the presence of effect modification unlikely.

This study adds to the growing list of examples of initial reports of effect modification in individual small studies that were subsequently refuted by large-scale evidence [27], a form of “winner's curse” [28]. Spurious effect modification is a concern when sample sizes are small [29]. In our analysis, we had over 10,000 CHD cases in over 130,000 individuals, meaning that the absence of evidence for heterogeneity in the estimates of *APOE* genotype with odds of CHD when stratified by smoking status indicates that a clinically relevant difference between smoking subgroups is very unlikely. Although it is tempting to interpret the estimate for CHD seen in non-smoking ε4 carriers as suggestive that this subgroup does not have an elevated risk of CHD compared to non-ε4 carriers (since the 95%CI includes the null), this is likely due to reduced power within strata of subgroups, and the salient feature is the lack of evidence for interaction between *APOE* genotype and smoking status.

It is curious that we did not replicate the presence of effect modification for two previous studies included in our analysis. This could be accounted for by updating one study (NPHSII) [6] for incident CHD events and in the other [9], effect modification was stronger in women than men. Furthermore, lack of access to individual participant data, and alternative analytical strategies in previous studies such as use of never smokers as the baseline group and comparing present and past smokers stratified by *APOE* genotype to this baseline, could also account for the discrepancy. It is noteworthy that both individual studies were relatively small and that the largest single study in this analysis (with a sample size over 6-fold greater than the combined sample size of the two previous studies with presence of effect modification) had the largest *P*-value for interaction.

There are several limitations to this study. Firstly, although we conducted a “quasi-individual participant data” analysis, we did not have access to individual participant data for all included studies, and therefore were not able to conduct multivariate analyses taking into account potential confounders such as age, gender and social status. However, we were able to conduct a detailed analysis in the large data sets that we had access to individual data. Second, we did not have markers of oxidative stress that we could use for a more detailed investigation into oxidative pathways as potential mediators of the association between *APOE* genotype and risk of CHD.

Our study also has several advantages. First, this is the largest analysis to date, incorporating new data from three very large prospective cohorts and five other studies. Second, using the data sets with refined data on *APOE* genotype status and in which outcome ascertainment was conducted using ICD codes to make our outcome specific to myocardial infarction, we were able to conduct a more detailed analysis by investigating evidence for a difference in the slope of the *APOE*–CHD relationship by smoking status. Third, we were able to examine a more detailed smoking phenotype (pack years) in two large prospective cohorts with a combined sample size of 68,177. Both the simple and detailed genetic and smoking analyses failed to identify evidence to support the hypothesis that smoking status acts as an effect modifier of the relationship between *APOE* genotype with CHD risk.

In conclusion, in the largest analysis to date including new data from large population-based cohorts, we identified no evidence to support the hypothesis that smoking status modifies the association between *APOE* genotype and risk of CHD. Regardless of these findings, as the leading cause of preventable disease and death in the world, smoking should be actively discouraged in all individuals.

## Financial disclosure

The funders had no role in study design, data collection and analysis, decision to publish, or preparation of the manuscript.

## Conflict of interest

The authors report no relationships that could be construed as a conflict of interest.
