# Supplementary material for: A systematic review and meta-analysis of 130,000 individuals shows smoking does not modify the association of APOE genotype on risk of coronary heart disease
Source: Atherosclerosis. 2014 Nov;237(1):5–12. doi: 10.1016/j.atherosclerosis.2014.07.038 (PMC4232362; doi:10.1016/j.atherosclerosis.2014.07.038)
Supplement: Supplementary file 1 [file mmc1.pdf]

SUPPLEMENTAL MATERIAL

Supplementary Methods

PubMed Search

We searched PubMed using the following search string:

("APOE" OR "apolipoprotein E*" OR "Apolipoproteins E"[MeSH]) AND ("smoking*" OR "smoke*" OR "tobacco*" OR "cigarette*" OR "Smoking"[MeSH] OR "Smoke"[MeSH] OR "Tobacco Use Cessation"[MeSH]) AND ("cardiovascular disease*" OR "coronary disease*" OR "Coronary Disease"[MeSH] OR "Coronary Artery Disease"[MeSH] OR "heart disease*" OR "CHD*" OR "Cardiovascular Diseases"[MeSH]) AND ("Genotype"[MeSH] OR “Alleles”[MeSH] OR “Polymorphism, Genetic”[MeSH] OR genotype* OR gene OR allele* OR polymorphism* OR genetic*)

EMBASE search

We used the following search string for EMBASE:

(APOE OR apolipoprotein E$ OR Apolipoproteins E) AND (smoking$ OR smoke$ OR tobacco$ OR cigarette$) AND (cardiovascular disease$ OR heart disease$ OR coronary disease$ OR CHD$ OR cardiovascular diseases) AND (Genotype OR Alleles OR Polymorphism OR Genetic OR genotype$ OR gene OR allele$ OR polymorphism$ OR genetic$)

Supplementary Table 1. PRISMA table

Section/topic	#	Checklist item	Reported on page #
**TITLE**			
Title	1	Identify the report as a systematic review, meta-analysis, or both.	1
**ABSTRACT**			
Structured summary	2	Provide a structured summary including, as applicable: background; objectives; data sources; study eligibility criteria, participants, and interventions; study appraisal and synthesis methods; results; limitations; conclusions and implications of key findings; systematic review registration number.	3
**INTRODUCTION**			
Rationale	3	Describe the rationale for the review in the context of what is already known.	4
Objectives	4	Provide an explicit statement of questions being addressed with reference to participants, interventions, comparisons, outcomes, and study design (PICOS).	5
**METHODS**			
Protocol and registration	5	Indicate if a review protocol exists, if and where it can be accessed (e.g., Web address), and, if available, provide registration information including registration number.	6
Eligibility criteria	6	Specify study characteristics (e.g., PICOS, length of follow-up) and report characteristics (e.g., years considered, language, publication status) used as criteria for eligibility, giving rationale.	6
Information sources	7	Describe all information sources (e.g., databases with dates of coverage, contact with study authors to identify additional studies) in the search and date last searched.	6
Search	8	Present full electronic search strategy for at least one database, including any limits used, such that it could be repeated.	6
Study selection	9	State the process for selecting studies (i.e., screening, eligibility, included in systematic review, and, if applicable, included in the meta-analysis).	6

Data collection process	10	Describe method of data extraction from reports (e.g., piloted forms, independently, in duplicate) and any processes for obtaining and confirming data from investigators.	6
Data items	11	List and define all variables for which data were sought (e.g., PICOS, funding sources) and any assumptions and simplifications made.	6
Risk of bias in individual studies	12	Describe methods used for assessing risk of bias of individual studies (including specification of whether this was done at the study or outcome level), and how this information is to be used in any data synthesis.	6
Summary measures	13	State the principal summary measures (e.g., risk ratio, difference in means).	8
Synthesis of results	14	Describe the methods of handling data and combining results of studies, if done, including measures of consistency (e.g., I^2) for each meta-analysis.	8
Risk of bias across studies	15	Specify any assessment of risk of bias that may affect the cumulative evidence (e.g., publication bias, selective reporting within studies).	8
Additional analyses	16	Describe methods of additional analyses (e.g., sensitivity or subgroup analyses, meta-regression), if done, indicating which were pre-specified.	8-9
**RESULTS**			
Study selection	17	Give numbers of studies screened, assessed for eligibility, and included in the review, with reasons for exclusions at each stage, ideally with a flow diagram.	11
Study characteristics	18	For each study, present characteristics for which data were extracted (e.g., study size, PICOS, follow-up period) and provide the citations.	23-25
Risk of bias within studies	19	Present data on risk of bias of each study and, if available, any outcome level assessment (see item 12).	11-12
Results of individual studies	20	For all outcomes considered (benefits or harms), present, for each study: (a) simple summary data for each intervention group (b) effect estimates and confidence intervals, ideally with a forest plot.	11-12
Synthesis of results	21	Present results of each meta-analysis done, including confidence intervals and measures of consistency.	11-12
Risk of bias across studies	22	Present results of any assessment of risk of bias across studies (see Item 15).	11-12
Additional analysis	23	Give results of additional analyses, if done (e.g., sensitivity or subgroup analyses, meta-regression [see Item 16]).	12-13
**DISCUSSION**			
Summary of evidence	24	Summarize the main findings including the strength of evidence for each main outcome; consider their relevance to key groups (e.g., healthcare providers, users, and policy makers).	14-15

Limitations	25	Discuss limitations at study and outcome level (e.g., risk of bias), and at review-level (e.g., incomplete retrieval of identified research, reporting bias).	15
Conclusions	26	Provide a general interpretation of the results in the context of other evidence, and implications for future research.	16
**FUNDING**			
Funding	27	Describe sources of funding for the systematic review and other support (e.g., supply of data); role of funders for the systematic review.	21

Supplementary Table 2. Proportion of *APOE* ε4 carriers and present smokers in studies identified by the systematic review.

First Author, Study	*APOE* ε4 carriers (%)	Present smokers (%)
Gustavsson J et al, / INTERGENE and SHEEP	27.58	25.17
Keavney et al, ISIS	28.14	38.69
Liu et al, Physicians' Health Study	25.72	15.60
Talmud et al, Whitehall II	25.84	7.27
Humphries et al, NPHSII	25.89	27.95
MRC GP Research Framework Investigators, TPT trial	29.20	40.15
ELSA	25.64	11.00
EPIC-Netherlands	25.32	24.66
EPIC-Norfolk	26.55	24.66
Copenhagen General Population Study (CGPS)	29.27	20.98
Copenhagen City Heart Study (CCHS)	28.62	47.45
Czech post-MONICA	17.71	22.03
HAPIEE-Czech	20.27	25.22

Supplementary Table 3. Estimates of linear trend between *APOE* genotype and CHD risk overall and stratified by smoking in the three large population-based cohorts.

	Cases/Total	Slope per unit increase in *APOE* genotype †, beta (standard error)	P value	P-value for heterogeneity
All individuals	6,334 / 89,706	0.046 (0.019)	0.017	N/A
**Smoking Status**				
Never smoked	1,687 / 35,826	0.034 (0.036)	0.345	0.352
Past smoker	3,158 / 34,869	0.076 (0.027)	0.005	
Present smoker	1,489 / 19,011	-0.002 (0.040)	0.954	

Footnotes: † *APOE* genotype arranged in the following order: ε2/ε2, ε2/ε3, ε3/ε3, ε3/ε4 or ε4/ε4. The slope represents the difference in log odds for a unit increase in *APOE* genotype. The three cohorts were CCHS, CGPS and EPIC-Norfolk (details in Table 1).

Supplementary Table 4. Association of *APOE* genotype with myocardial infarction (MI) risk stratified by pack years in Copenhagen General Population Study (n=59,349).

Smoking Status (pack years)	CHD cases/Total	OR of MI for *APOE* genotype (ε4 carrier vs non-carrier), OR (95%CI)	P value	P-value (test for interaction)
0	565/24,867	0.89 (0.75, 1.08)	0.26	0.54
>0 to <10	272/11,590	1.07 (0.82, 1.38)	0.64	
≥10 to <20	336/7,748	1.00 (0.78, 1.27)	0.97	
≥20	1,152/15,144	1.06 (0.93, 1.20)	0.42	

Supplementary Table 5. Association of *APOE* genotype with myocardial infarction (MI) risk stratified by pack years in Copenhagen City Heart Study (n=8,828).

Smoking Status (pack years)	MI cases/Total	OR of MI for *APOE* genotype (ε4 carrier vs non-carrier), OR (95%CI)	P value	P-value (test for interaction)
0	177/2,426	0.80 (0.56, 1.14)	0.21	0.65
>0 to <10	68/1,410	1.08 (0.64, 1.82)	0.76	
≥10 to <20	142/1,241	0.97 (0.65, 1.44)	0.87	
≥20	728/3,751	1.01 (0.85, 1.21)	0.88	

Supplementary Table 6. Tests for interaction between *APOE* genotype and pack years for risk of myocardial infarction in Copenhagen General Population Study (n=59,349) with adjustment for cardiovascular traits.

**Covariate**	**LRT P-value**
Unadjusted	0.54
Age (10-yr age bands; mean age=56yrs)	0.49
Gender (33,108 men, 26,241 women)	0.55
T2D (2,059 cases, 57,290 non-cases)	0.61
Hypertension (34,398 cases, 24,951 non-cases)	0.48
All covariates	0.61

LRT: Likelihood ratio test

Supplementary Table 7. Tests for interaction between *APOE* genotype and pack years for risk of myocardial infarction in Copenhagen City Heart Study (n=8,828) with adjustment for cardiovascular traits.

Covariate	LRT P-value
Unadjusted	0.65
Age (10-yr age bands; mean age=55yrs)	0.75
Gender (4931 men, 3897 women)	0.64
T2D (8110 cases, 718 non-cases)	0.64
Hypertension (4366 cases, 4462 non-cases)	0.67
All covariates	0.71

LRT: Likelihood ratio test

Supplementary Figure 1 PRIMSA flow diagram

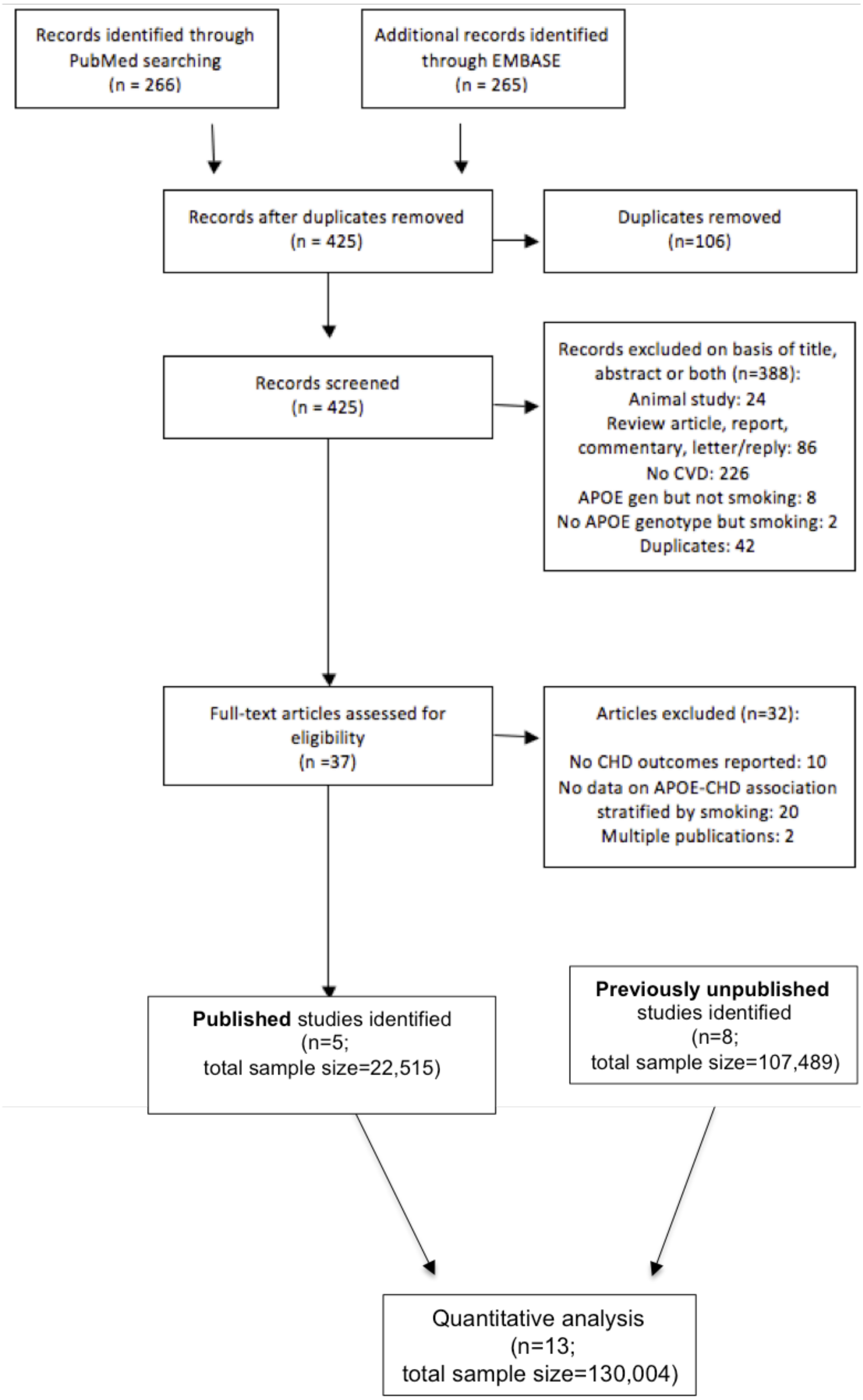

Supplemental Text

UNIVERSITY COLLEGE LONDON

MSc Pharmacogenetics and Stratified Medicine

PROJECT PLAN
2011-2012

Project title:

Meta-analysis of interaction between APOE genotype and smoking on CHD risk.

Supervisors: Philippa J. Talmud and Michael Holmes.

Candidate: Daniela Melis

Coronary heart disease (CHD) is a major cause of mortality in the world and is known to be modified by the interaction of functional gene polymorphism and environmental factors[1]. Smoking is well known to be one of the most important environmental factors associated with APOE genotype on CHD risk. In this project we are focusing on assess the role of the APOE genotype and smoking on CHD risk.

APOE gene codifies the apolipoprotein (Apo) E which is one of five main types (A, B, C, D, and E) of apolipoproteins; that together with phospholipids forms the external layer of the plasma lipoproteins. ApoE helps to stabilize and solubilize lipoproteins as they circulate in the blood. In general, the role of apolipoproteins in lipid metabolism includes maintaining the structural integrity of lipoproteins, serving as cofactors in enzymatic reactions, and acting as ligands for lipoprotein receptors. Apo E is critical in the formation of very low density lipoprotein (VLDL) and chylomicrons. It is synthesized primarily in the liver, but other organs and tissues also synthesize it, including brain, spleen, kidneys, gonads, adrenals, and macrophages[2].
The APOE gene is located in on the long arm of chromosome 19 at position 13.2 (19q13.2), which consists of four exons and three introns spanning 3,597 nucleotides and produces the 299 amino acid polypeptide with a molecular weight of 34 KDa. The structural gene is polymorphic with three common co-dominant alleles, ε2, ε3, and ε4, producing three isoforms of the protein, E2, E3, and E4[1]. ε3 is the most common allele with a frequency of 75–80% in most populations[3]. From these alleles arise six phenotypes; their ranking from most to least common is generally 3/3, 4/3, 3/2, 4/4, 4/2, and 2/2[1]. The gene frequencies among different populations demonstrate

to have a geographic cline. Northern Europeans (Finns, Germans) tend to have higher frequencies (~14–19 percent) of the ε4 allele than southern Europeans (French, Italians) (~7–12 percent). Nigerians, Japanese, and Finns have relatively low frequencies (~3–4 percent) of ε2. Mexican Americans and American Indians also have low frequencies (~2–4 percent) of the ε2 allele[2].
These isoforms differ in amino acid sequence at positions 112 and 158. Apo E3 contains cysteine at 112 and arginine at 158. Apo E2 has cysteine at both positions, and E4 has arginine at both sites[4].
The apoE gene polymorphism has a strong effect on the level of its gene product; ε2 is associated with higher concentrations of apo E and ε4 with lower concentrations. The three isoforms differ, also, in their low density lipoprotein (LDL)-receptor affinity, antioxidant activity (E2 > E3 > E4) and inflammation modulatory properties[3]. The various apoE isoforms interact differently with specific lipoprotein receptors, ultimately altering circulating levels of cholesterol. Apo E from VLDL and chylomicron remnants binds to specific receptor cells in the liver. Carriers of the ε2 allele are less efficient at making and transferring VLDLs and chylomicrons from the blood plasma to the liver because of its binding properties. By contrast, carriers of the ε3 and ε4 alleles are much more efficient in these processes. While Apo E4 and E3 bind with approximately equal affinity to lipoprotein receptors, apoE2 binds with less than 2 percent of this strength. Thus, compared with carriers of the ε3 or ε4 allele, carriers of the ε2 allele are slower to clear dietary fat from their blood. The difference in uptake of postprandial lipoprotein particles results in differences in regulating hepatic low density lipoprotein (LDL) receptors, which in turn contributes to genotypic differences in total and LDL cholesterol levels[2].

While there are rare variants, it is the polymorphism with its three alleles, ε2, ε3, and ε4, which has been studied quite extensively in relation to cardiovascular disease[3]. In many studies APOE alleles have been shown to influence the risk of cardiovascular diseases[2]. It is established that APOE genotype have an approximately linear relationship with low density lipoprotein cholesterol and CHD risk when ordered ε2ε2, ε2ε3, ε2ε4, ε3ε3, ε3ε4, ε4ε4 [4]. However, there is evidence from a number of studies that in no smokers APOE genotypes have no effect on CHD, but in individuals who smoke there is significant evidence that ε4 carriers show a greater risk compared to ε3 homozygotes smokers and non-smokers, while ε2 carriers were protected from risk[5, 6, 7]. In fact, people who carry at least one copy of the APOE ε4 allele have an increased chance of developing atherosclerosis, which causes increase risk of heart attack and stroke. Smoking makes a great contribution on onset of coronary heart disease. The product of tobacco combustion directly damage vascular endothelium, which promoting thrombosis and atherosclerosis. Also, smoking can increase risk of thrombosis because it induces lung damage and a consequent inflammatory process. Furthermore, smoking is implicated in production of small dense LDL-cholesterol and smokers have lower circulating concentration of antioxidants such as ascorbate and tocoferol than non-smokers, which might favour oxidation of LDL[1]. The most likely mechanism to explain the e4: smoking interaction on CHD risk is just through a direct effect on LDL oxidation[6]. As it has been demonstrated in vitro, the three isoforms of APOE have differential oxidation with apoE4 being more susceptible than E3, which in turn is more susceptible than apoeE2 to oxidation. This effect may be due to the fact that ApoE2 has 2 free SH-groups, ApoE3 has 1and ApoE4 none, or maybe due to other effects of ApoE isoforms on the physico-chemical properties of lipoproteins that promote or protect from oxidation. Some studies, however, do not confirm the

interaction between APOE ε4 allele and CHD risk[8]. For that reason the APOE: smoking interaction on CHD risk needs further validation. The aim of this project is just to try and resolve this by meta-analysis of published data and by making contact with study leaders of published and ask for re-analysis of existing APOE CHD data after stratification by smoking. Pool results of existing researches will allow obtaining valid conclusion, maximizing power and minimizing bias of data results.

Methods:

Electronic searches of published data have been performed using MEDLINE and EMBASE database, using the following index terms: *APOE, Apolipoproteins E, smoking, smoke, tobacco, cigarettes, cardiovascular disease, heart disease, coronary heart disease, CHD, Genotype, Alleles, Polymorphism, Genetic, gene, allele, or polymorphism*. This search leaded to a total of 339 different articles. All studies were considered potentially eligible if they aimed to investigate the relation between ApoE genotypes, smoking and CHD risk; excluding review papers, comments and editorials. From a first selection, the number of relevant articles fell at 156. Further analysis of these papers will be undertaken to select each included study that clearly describes the study design, the control section, the CHD phenotype, genotype frequency, smokers frequency and genotyping methods. Also, the included studies should reported the relative risks or ORs and 95% CI for CHD related to apoE polymorphism and smoking. Subsequently, a statistical analysis of the selected data will be performed using STATA 11.1 software package[9].

References

1. Talmud PJ (2007). Gene-environment interaction and its impact on coronary heart disease. Nutr Metab Cardiovasc Dis.;17(2):148-52.
2. Eichner JE, Dunn ST, Perveen G, Thompson DM, Stewart KE and Stroehla BC. Apolipoprotein E Polymorphism and Cardiovascular Disease: A HuGE Review. Am J Epidemiol., 2002,**155**(6):487-95.
3. Gustavsson J, Mehlig K, Leander K, Strandhagen E, Björck L, Thelle DS, Lissner L, Blennow K, Zetterberg H and Nyberg F. Interaction of apolipoprotein E genotype with smoking and physical inactivity on coronary heart disease risk in men and women. Atherosclerosis, 2012, **220**(2):486-92.
4. Bennet AM, Di Angelantonio E, Ye Z, Wensley F, Dahlin A, Ahlbom A, Keavney B, Collins R, Wiman B, de Faire U and Danesh J. Association of apolipoprotein E genotypes with lipid levels and coronary risk. JAMA, 2007, **298**(11):1300-11.
5. Talmud PJ, Lewis SJ, Hawe E, Martin S, Acharya J, Marmot MG, Humphries SE and Brunner EJ. No APOEepsilon4 effect on coronary heart disease risk in a cohort with low smoking prevalence: the Whitehall II study. Atherosclerosis, 2004, **177**(1):105-12.
6. Humphries SE, Talmud PJ, Hawe E, Bolla M, Day IN and Miller GJ. Apolipoprotein E4 and coronary heart disease in middle-aged men who smoke: a prospective study. Lancet, 2001, **358**(9276):115-9.
7. Talmud PJ, Stephens JW, Hawe E, Demissie S, Cupples LA, Hurel SJ, Humphries SE and Ordovas JM. The significant increase in cardiovascular disease risk in APOEepsilon4 carriers is evident only in men who smoke: potential relationship between reduced antioxidant status and ApoE4. Ann Hum Genet, 2005, **69**:613-22.
8. Keavney B, Parish S, Palmer A, Clark S, Youngman L, Danesh J, McKenzie C, Delépine M, Lathrop M, Peto R and Collins R. Large-scale evidence that the cardiotoxicity of smoking is not significantly modified by the apolipoprotein E ε2/ ε3/ ε4 genotype. Lancet, 2003, 361:396–8.
9. StataCorp. 2009. Stata Statistical Software: Release 11. College Station, TX: StataCorp LP.
